# A new green method to NiO/MnO_2_ nanocomposite synthesis for efficient and sustainable dye removal from aqueous media

**DOI:** 10.1038/s41598-025-30677-z

**Published:** 2025-12-27

**Authors:** Ibrahem M. A. Hasan, Hassan M. A. Salman, Olfat M. Hafez

**Affiliations:** Chemistry Department, Faculty of Science, South Valley University, Qena, 83523 Egypt

**Keywords:** Adsorption, Biosynthesis, *Ficus benjamina*, Malachite green, Nickel oxide/manganese oxide nanocomposite, Chemistry, Environmental sciences, Materials science, Nanoscience and technology

## Abstract

The bimetallic nickel oxide/manganese oxide nanocomposite (NiO/MnO_2_ NC) was prepared for the first time using a one-pot plant-mediated route and applied for the adsorption of malachite green (MG) dye from aqueous media compared with the monometallic nickel oxide nanoparticles (NiO NPs). The materials were characterized by TGA, FTIR, XRD, EDX, SEM, TEM, and BET analyses. The adsorption parameters, including pH, MG initial concentration, agitation time, adsorbent mass, and temperature, were systematically studied. The results indicated a significant effect of all parameters on the MG removal percent, attaining 90.4% by NiO NPs and 99.64% by NiO/MnO_2_ NC within 60 min. The experimental data are best fitted by the Freundlich model for NiO NPs and the Langmuir model for NiO/MnO_2_ NC, having maximum adsorption capacities of 39.84 and 208.3 mg/g, respectively. This can be attributed to the higher surface area of NiO/MnO_2_ NC (143.65 m^2^/g) than NiO NPs (3.75 m^2^/g), as well as the presence of different metal atoms in the nanocomposite, which form unique adsorption sites with variable energies, thereby improving adsorption. Although the pseudo-second-order kinetic model more accurately describes kinetics, isotherm models, particularly the D-R isotherm model, show that physisorption is the primary mechanism. The combined data point to a multi-mechanistic adsorption process, with physisorption being the primary mechanism.The thermodynamic findings displayed that MG adsorption process is spontaneous, feasible, and endothermic. Both adsorbents are stable after five adsoption cycles, and the MG adsorption is mainly due to electrostatic attraction, hydrogen bonds, and π-π stacking. These results conclude that NiO/MnO_2_ NC is a better alternative to NiO NPs for removing MG, providing insights into designing more efficient adsorbents for dye removal in the water treatment systems.

## Introduction

 Industries discharge significant amounts of chromatic dyes, heavy metals, pharmaceutical residues, and other organic pollutants leading to water resources contamination^1^. Synthetic dyes are detected in greater environmental concentrations because of technological advancements in industry, human activity, and their low removal rate during waste purification^2^. Around 10% to 15% of the dye produced worldwide is lost to wastewater via different processes, including congo red, methylene blue, crystal violet, and malachite green^3^. Among these synthetic dyes, malachite green (MG) is widely used and abundant. MG is an example of heterocyclic aromatic chemicals that negatively affect the environment and people^4^. As an essential cationic dye, MG has been widely used for coloring leather, wool, and silk, as well as in distilleries. Additionally, MG is employed in the aquaculture sector as a fungicide and an antiseptic to manage fish parasites and diseases. However, reports on animal studies have revealed that MG has carcinogenic, genotoxic, mutagenic, and teratogenic effects because of nitrogen^5^. Because of this, the difficulty of removing MG from wastewater has grown significantly. Despite this, residual MG from wastewater is frequently challenging to remove due to its chemical stability, complicated disintegration, and presence in living things and the natural environment^6^.

Several techniques have been used to develop methods for eliminating MG from wastewater, including advanced oxidation^6^, biotransformation^7^, coagulation sedimentation^8^, membrane filtration^9^, chemical oxidation^10^, photocatalytic degradation^11^, aerobic and anaerobic microbial treatments^12^, and adsorption^13–15^. Nevertheless, several of these techniques have drawbacks, such as the need for chemicals, their poor effectiveness, and the fact that they typically generate significant amounts of sludge, which can exacerbate existing environmental issues^16^. Adsorption is now recognized as a highly promising physicochemical method for removing MG found in wastewater owing to its high efficiency, simplicity of use, low cost, and wide range of applications^11^.

The most often used adsorbents include activated carbons, clays, zeolites, biodegradable polymers, and synthetic polymer sorbents; although, these adsorbents may have limited adsorption capabilities and separation issues^17–21^. However, metal oxide nanostructures and nanocomposites are a good fit for these requirements owing to surface functional groups that can interact with dyes and heavy metal ions and the large surface area. Thus, they demonstrate a fantastic potential for water treatment^22^.

Nickel oxide; NiO is a significant transition metal oxide with unique characteristics that make it useful in various fields, such as electrical, chemical, and optical properties^23^. It has been found that NiO nanoparticles (NPs) are an efficient adsorbent for metal ions and dyes. It has minimal manufacturing costs, natural porosity, ample surface space, high availability, and eco-friendly properties^24^. NiO NPs, a particularly interesting and promising functional oxide, have been found in wide applications as catalysts, lithium-ion batteries, supercapacitors, gas sensors, and magnetic and water treatment materials^25^.

Recently, researchers worldwide have looked at many methods to prepare relevant MnO NPs, including MnO_2_ NPs, Mn_2_O_3_ NPs, and Mn_3_O_4_NPs^26,27^. It is a potent antibacterial and antioxidant because it is a stable metal oxide with synergistic effects. Furthermore, MnO_2_ NPs have received a lot of attention because they are believed to have a lesser potential for cytotoxicity than other nanoparticles^28^. Compared to single metal nanoparticles, the dual metal nanostructure exhibited higher adsorption activity^29,30^. Many distinctive characteristics, such as chemical and thermal stability, nontoxicity, and cost-effectiveness, may be present in the resulting nanocomposite (NC) when MnO_2_ is mixed with NiO. However, dual nanocomposites have received little attention despite showing great potential in several applications^31^.

.

Although numerous studies report plant-mediated syntheses of the single metal oxides NiO and MnO₂ using leaf, flower, or fruit extractsthe literature shows that NiO/MnO₂ binary nanocomposites are overwhelmingly prepared by conventional routes rather than by a direct, one-pot plant-extract method. For example, several authors have demonstrated green syntheses of MnO₂ nanoparticles using plant extracts^26,27^. Similarly, plant-mediated NiO syntheses have been reported independently^32–34^. In contrast, representative NiO/MnO₂ composite studies typically employ hydrothermal or co-precipitation routes rather than a single phyto-synthetic approach^35^. While plant extracts have been used to produce other mixed systems such as Ag–MnO₂^28^, these serve primarily as methodological precedents rather than direct examples of a biogenic NiO/MnO₂ composite. Taken together, these observations indicate a clear gap: there are few (if any) reported protocols for the direct, one-pot plant-extract synthesis of NiO/MnO₂ nanocomposites, representing an opportunity to develop genuinely green compositional-control strategies and to benchmark their functional performance against conventionally prepared counterparts.

*Ficus benjamina* (*Moraceae* family) is a Southeast Asian evergreen tree known as the weeping fig. Traditional medicine has employed several portions of this plant to treat anti-dysentery and skin ailments^36^. Leaf extracts of FB trees showed a wealth of bioactive chemical components, including proteins, sugars, alkaloids, flavonoids, phenolic mixtures, and tannins^37^. To the best of our knowledge, we are the first to report the utilization of FB for the synthesis of NiO/MnO_2_ NC.

This study addresses both environmental and economic concerns by using agricultural waste to produce high-efficiency adsorbents, which is in line with the circular economy and waste-to-resource plans. It aims to green synthesize and characterize NiO NPs and NiO/MnO_2_ NC using FB leaves extract, assess and compare their dye adsorption efficiency via batch tests, and evaluate their regeneration and reusability potential for sustainable water treatment applications.

## Materials and methods

### Materials

All the chemicals and reagents employed in synthesizing NiO NPs and NiO/MnO_2_ NC are of analytical grade. Nickel (II) acetate tetrahydrate (Ni(CH_3_CO_2_)_2_.4H_2_O), KMnO_4_ (Purity ≥ 99%), MG (C_52_H_54_N_4_O_12_, purity ≥ 98%), crystal violet (CV) (C_25_N_3_H_30_Cl purity < 99%), and rhodamine B (RhB) (C_28_H_31_ClN_2_O_3_ purity < 99%) attained from Sigma Aldrich company. *FB* aqueous leaf extract was used as a reductant and stabilizing agent. Bidistillated water was utilized to prepare aqueous solutions.

### Preparation of FB leaf extract (FBLE)

Fresh leaves of *FB* were gathered from South Valley University during the winter season. At that point, they were distinguished and authenticated by the members of the Department of Botany. Fresh leaves were plucked from the branches, meticulously cleaned, and then chopped into tiny pieces after being rinsed three times with tap water and then again with deionized water to eliminate any potential mud and dust that may have accumulated on the plant material. 25 g of freshly cleaned *FB* leaves and 250 ml of distillate water (10% w/v) were combined to extract the aqueous leaves. After that, the resultant mixture was then heated on a hot plate at 80 °C for two hours and soaked overnight. After that, it was decanted and filtrated twice through Whatman filter paper (125 mm) at room temperature. Finally, the pale brown clear solution, which indicated the release of the polyphenolic compounds from the leaves, was collected and stored in the refrigerator at 4 °C for further use^14^.

### Green synthesis of the nanoadsorbents using FBLE

#### Preparation of NiO NPs

About 5 g of nickel acetate Ni(CH_3_COO)_2_·4H_2_O was dissolved in 150 ml bidistilled water. Then, the freshly prepared solution was added to 300 ml of FBLE with a volume ratio of 1:2. After that, a magnetic stirrer was used to stir the mixture vigorously for three hours at 70 °C. The reaction mixture’s color gradually changed until a black precipitate formed, which indicated the formation of NiO NPs. Then, the mixture was aged overnight to ensure the complete reduction of nickel ions. The reaction mixture was dried overnight in an oven at 120 °C. Then, the remaining residue was collected, washed several times with bidistilled water and ethanol, and dried in an oven at 80–90 °C overnight. The solid sample was calcined at 500 °C for three hours in a muffle furnace. Finally, the obtained greyish-black product was crushed using a pestle and mortar and stored in an airtight container for further use.

#### Preparation of NiO/MnO_2_ nanocomposite

NiO/MnO_2_ nanocomposite was synthesized by mixing 5 g KMnO_4_ and 5 g Ni(CH_3_COO)_2_.4H_2_O with a mass ratio of 1:1 in 150 bidistilled water. They stirred for 30 min until a clear solution was formed. Afterward, 300 ml of FBLE solution was added dropwise. The reaction mixture solution was kept under constant stirring for 3 h at 70 °C. The mixture was dried overnight in an oven at 120 °C. The resulting solid residue was washed several times with bidistilled water and ethanol, then kept at 90 °C overnight for drying and stored in bottles under dry conditions.

### Instrumentation for characterization of FBLE and the biosynthesized NPs

The biosynthesis of NPs employing *FBLE* was examined using a UV-visible spectrophotometer (PG Instruments, model T80, UK). The thermal stability was assessed by TGA (SDT Q600 V20.9 Build 20) with a 30 °C/min heating rate up to 600 °C under Ar gas flow at 100 ml/min. To figure out the potential contribution of phytochemicals present in *FBLE* to the synthesis of NPs, FTIR spectra were produced in the 400–4000 cm^− 1^ range using the KBr pellet method (Shimadzu FTIR, Kyoto, Japan). X-ray diffraction spectra were recorded using a powder diffractometer at room temperature to examine the phase structure and crystallite size of the biosynthesized sample (Brucker D8 Advance, Germany with Cu Kα radiation source, λ = 1.5406 Å and 2θ in the range (10–80°). The Scherrer equation was used to compute the average crystallite size of the produced samples (D = 0.9λ/(βcosθ)), where D is the average crystallite size (nm), λ is the X-ray wavelength used (λ = 1.54056 Å), θ is the diffraction angle, and β is the full width at half the maximum of the diffraction peak in radians. A scanning electron microscope was used to analyze the morphology, size, and chemical composition of the synthesized NPs (QUANTA FEG250) attached with energy dispersive X-ray (EDX; Inspect S 50, FEI, Netherlands), which was operated at 20 kV accelerating voltage, 10 mm working distance and probe current of 1.0 nA. To provide morphologic, compositional, and crystallographic information on the synthesized samples, TEM was carried out using Jeol Jem-1230 operating at a voltage of 200 KV for acceleration. BEL SORP-MAX analyzer (MicrotracBEL, Japan) was employed to measure the specific surface area (SSA). The SSA value was calculated using the multipoint BET (Brunauer–Emmet–Teller) method based on the N_2_-sorption data. Barrett–Joyner–Halenda (BJH) was adopted to measure the average pore diameter.

### Study of point of zero charge (PZC)

The point of zero charge (PZC) of NiO NPs and NiO/MnO_2_ NC was obtained by the pH drift method^25^ with some modifications. Briefly, fixed sorbent loading of 2.0 g/l was combined with 50 ml of an aqueous NaCl (0.01 M) solution in a 250 ml reagent bottle, and then NaOH (0.1 M) or HCl (0.1 M) solutions were used to change the initial solution pH (pH_i_) in each bottle at pH ranges (2–10). After that, the mixtures were agitated with the sorbent for 48 h to reach equilibrium. Finally, the final pH of the solutions (pH_f_) was measured. ΔpH was calculated (ΔpH = pH_i_ – pH_f_) and plotted versus pH_i_. The PZC will be the point that reaches the null value variation (ΔpH = 0). Before every run, the pH meter was calibrated at pH values 2, 5, 7, and 10^14^.

### Optimization of MG adsorption

Adsorption experiments were carried out using a batch approach. The initial pH, adsorbent dosage, contact times, solution temperature, and solution concentration were among the examined parameters. In the batch pH studies procedure, 0.05 g of NiO with 50 ml of 10 mg L^− 1^ MG were combined and agitated at 250 rpm at 298 K for 60 min using a temperature-controlled shaker. Concentrations were determined using a UV–Vis spectrophotometer (PG Instruments, model T80, UK) at 617 nm. Dilute NaOH or HCl solutions were used to change the solutions’ initial pH values to a range of levels (3, 5, 7, 9, and 10). pH measurements were conducted using a pH meter (Mettler Toledo S220, Columbus, OH).

Additionally, batch experiments were conducted at 298 K and the optimal pH for the solution during a 60-minute shaking period to investigate the impact of adsorbent dosage. Various quantities of NiO NPs and NiO/MnO_2_ NC (ranging from 0.01 to 0.1 g and 0.001 to 0.01 g, respectively) were introduced into each 50 ml MG solution. The following formula was applied to calculate the elimination percent of MG:

1 $$\:{R}{\%}\:=\left(\frac{{{C}}_{{o}}-\:{{C}}_{{e}}}{{{C}}_{{o}}}\:\right).\:100$$

where (C_o_ and C_e_ mg/l) are the initial and equilibrium concentrations of the dye (MG), respectively. To determine the adsorption isotherm and examine the impact of initial concentration on MG removal, a 50 ml MG solution with varying initial concentrations (5–50 mg/l) and temperatures (298, 308, 318, and 328 K) at pH 10 was introduced into contact with 50 mg of NiO NPs and 10 mg of NiO/MnO_2_ NC. The flasks were agitated at 250 rpm for 60 min until equilibrium was reached. A UV spectrophotometer was employed to determine the amounts of MG in the suspensions after they had been centrifuged for five minutes at 5000 rpm. Using the following formula, the equilibrium uptake of MG, represented as q_e_ (mg/g), was calculated:

2 $$\:{{q}}_{{e}}\:=\:\left({{C}}_{{o}}\:-\:{{C}}_{{e}}\right).\frac{{V}}{{W}}$$

where C_o_ and C_e_ (mg/l) are identified above, V (L) is the MG solution volume, and W (g) is the weight of the adsorbent used. The equilibrium data were fitted with the Langmuir, Freundlich, Dubinin–Radushkevick (D-R), and Temkin isotherm models. The effect of agitation time on MG adsorption was tested by mixing 50 ml MG aqueous solutions (10 mg/l) with 50 mg of NiO NPs at different time intervals ranging from 20 to 100 min. The adsorption of MG by NiO/MnO_2_ NC was studied by using 50 mg/l dye concentration at varying times (15–60 min). The adsorption kinetics were determined by applying the intra-particle diffusion (IPD), pseudo-first order (PFO), pseudo-second order (PSO), and Elovich models to the collected data. Three thermodynamic parameters change in the Gibbs free energy (ΔG°), enthalpy (∆H°), and entropy (∆S°) were determined to evaluate the thermodynamic feasibility and the nature of the adsorption process.

### Interfering ions

The effect of sodium and calcium interfering ions has also been studied. The concentration of these ions was 2, 4, 6, 8, and 10 g/l under the experimental conditions of 50 ml MG solution, 10 mg/l MG concentration, 10 mg NiO NPs mass, and 50 mg NiO/MnO_2_ NC mass. The mixtures of MG and interferences were stirred for 60 min at 200 rpm speed and pH 10 at room temperature (298 K).

### Mixed dyes studies

The influence of the coexistence of other cationic dyes, crystal violet dye (CV: λ_max_ 586 nm) and rhodamine B (RhB: λ_max_ 558 nm), on the MG adsorption was examined at optimum conditions. In each experiment, 50 ml, 10 mg/l solution of each of MG and interfering dye is mixed and stirred for 60 min at 200 rpm speed and pH 10 at room temperature (298 K).

### Stability and reusability

Cost-effective adsorbents must be stable and reusable. In this regard, five consecutive runs were conducted to investigate the regeneration and reuse of NiO NPs and NiO/MnO_2_ NC for MG dye adsorption. During the first run, 50 ml of 10 mg/l MG solution and 1 g/l NiO NPs or 0.2 g/l NiO/MnO_2_ NC were mixed for 60 min at pH 10. The adsorbent was then separated by centrifugation for five minutes at 5000 rpm. As previously stated, the supernatant is analyzed using a UV–Vis spectrophotometer to determine the remaining MG. Finally, the gathered adsorbent was washed three times with a 1:1 mixture of bidistilled water and ethanol, then dried in an electric oven for two hours at 80 °C before being introduced to the next run.

## Results and discussion

### Characterization of *FBLE* and the biosynthesized NiO NPs and NiO/MnO_2_ NC

#### UV–Vis

**Fig. 1:**
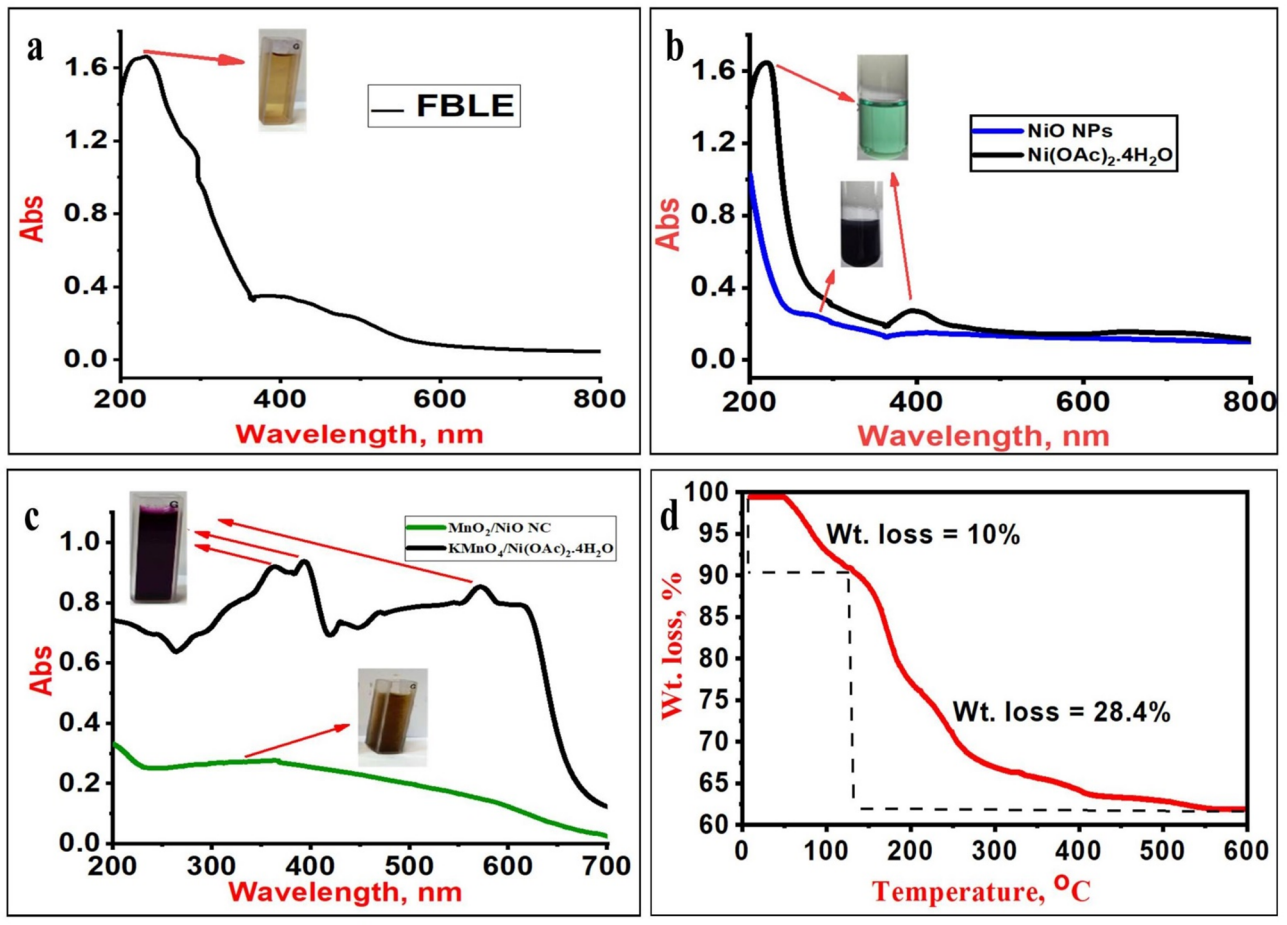
UV–Vis spectra of FBLE (**a**), nickel acetate solution; black line, and synthesized NiO NPs; blue line (**b**), potassium permanganate/nickel acetate solution; black line, and synthesized NiO/MnO_2_ NC; green line (**c**), and TGA curve of the synthesized NiO NPs (**d**).

Figure 1a shows the UV–Vis spectrum of FB extract. The FB leaf extract exhibits a characteristic peak at 231 nm, attributed to the polyphenolic content^38^. Figure 1b displays the UV–Vis spectra of nickel acetate and the biosynthesized NiO NPs. The nickel acetate spectrum (black line) has two peaks at 390 and 220 nm. The absence of these peaks in the NiO NPs spectrum (blue line) indicated the reaction of polyphenols with nickel acetate. The maximum absorption peak, characteristic of NiO NPs, was located at 285 nm^32,39,40^.

Figure 1c shows the UV–Vis adsorption spectra of KMnO_4_/Ni(OAc)_2_·4H_2_O precursor (black line) and the synthesized NiO/MnO_2_ NC (green line). The precursor shows absorption peaks at 320, 390, and 570 nm. The characteristic peak 290 nm is observed in the UV–Vis spectrum of the NiO/MnO_2_ NC, where a red shift can be observed due to the lattice distortion and the introduction of new localized bands^41^. Interestingly, in NiO/MnO_2_ NC, only a broad peak is observed at the wavelength range of 290–320 nm owing to the coupling between the absorption peaks of MnO_2_ NPs and NiO NPs.

#### TGA

Figure 1d shows the TGA analysis results. The initial constant 10% weight loss was seen within the 50 to 130 °C temperature range, and this is attributed to the vaporization of H_2_O molecules that were absorbed on the surface of the NiO NPs. At a temperature range of 130–500 °C, a second significant loss with a proportionate amount of 28.4% was observed. This loss was most likely caused by the decomposition of organic molecules covering the NiO NPs^42^. Finally, above 500 °C, no significant weight loss is observed, which implies that the nickel oxide formation is complete.

#### FTIR

**Fig. 2:**
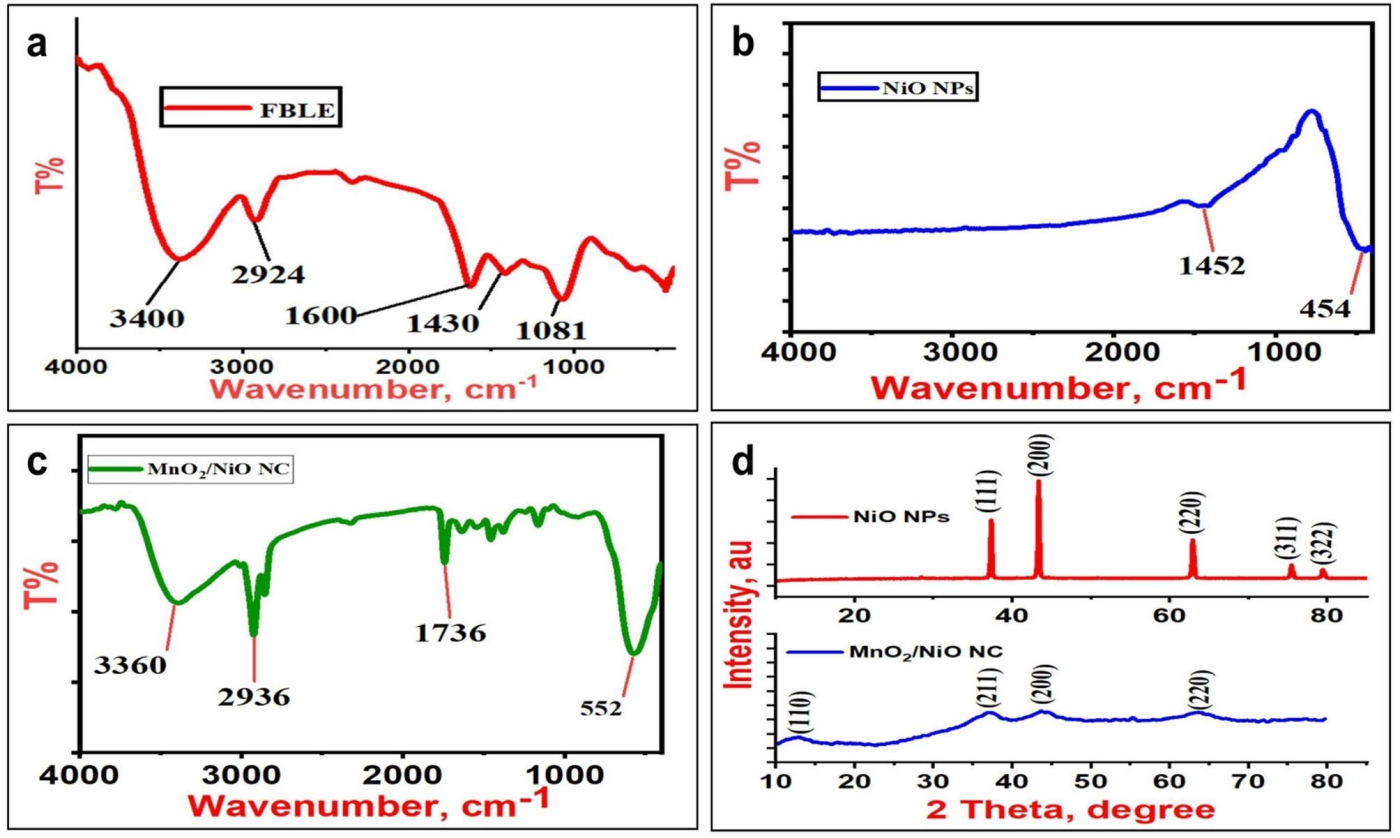
FTIR spectra of FBLE (**a**), the synthesized NiO NPs (**b**) and NiO/MnO_2_ NC (**c**), and the XRD patterns of NiO NPs; red line and NiO/MnO_2_ NC; blue line (**d**).

The FTIR spectra of FBLE, NiO NPs and NiO/MnO_2_ NC were obtained within the 4000–400 cm^− 1^ range. The FTIR spectrum of FBLE is shown in Fig. 2a and exhibits several absorption bands at 3400, 2924, 1600, 1430, and 1081 cm^− 1^ that are assigned to O-H stretching vibration, C–H stretching vibration, aromatic C=C bond stretching, methylene group C–H bending, and C–O stretching, respectively^38,43^. The NiO NPs spectrum (Fig. 2b) showed a peak at 1452 cm^− 1^ belonging to the oxide groups^22^ and a peak at ~ 454 cm^− 1^, which is caused by the Ni-O bending vibration^44,45^. Calcination resulted in the removal of most FB phytochemicals from NiO NPs. On the other hand, NiO/MnO_2_ NC is not calcined and thus its FTIR spectrum (Fig. 2c) shows peaks at 3360 and 2936 cm^− 1^ corresponding to the O–H stretching vibration, confirming hydrogen-bonded hydroxyl groups^46^. The peak at 1736 cm^− 1^ represents to the interlayer water molecules’ O–H bending vibration^47^. The broad peak in the 552–620 cm^− 1^ range combines Mn–O and Ni–O vibrations^48^. Noticeably, the characteristic peak at 552 cm^− 1^ for the MnO_2_ NPs is observed in the nanocomposite, proving the successful synthesis of the NiO/MnO_2_ NC^49^. In this approach, it can be observed that phytochemicals presented in plants are essential as reducing agents in the formation of nanoparticles^50^.

#### Powder XRD

XRD analysis was employed to estimate the phase structure and crystallite size of nanoparticles. The XRD pattern in Fig. 2d demonstrated the formation of NiO NPs and NiO/MnO_2_ NC. The planes (111), (200), (220), (311), and (222) can be attributed to the peaks at 2θ values of 37.27°, 43.08°, 62.42°, 74.94°, and 78.95°, respectively. A pure cubic phase of NiO with a = b = c = 4.197 was readily identified by all of the diffraction peaks (JSPDS Card no. 65-2901). Sharp diffraction peaks confirm the high crystallinity of the produced nanoparticles, and the absence of impurity peaks indicates the high purity of monophasic NiO. Debye–Scherrer formula was utilized to calculate the mean crystallite size of the synthesized NiO NPs, which was 37 nm as shown in Table 1.

The XRD of the NiO/MnO_2_ NC. The peaks at 2θ 12.74° and 37.62° that correspond to (110) and (211) planes are attributed to MnO_2_ NPs (COD card No.90-16667)^14^. However, the peaks at 2θ 43.08° and 62.42° that correspond to (200) and (220) planes are attributed to NiO NPs (JCPDS card no. 43-20490). This confirms the successful formation of NiO/MnO_2_ NC. Furthermore, the diffraction peaks of MnO_2_ and NiO are mainly broadened, and their intensities are decreased, confirming a much smaller crystallite size than the pure oxides. The mean crystallite size of the NiO/MnO_2_ NC was calculated as 9.84 nm, which is about four times smaller than NiO NPs (Table 1).

**Table 1:** Main properties of the synthesized nanoadsorbents.

Property	NiO NPs	NiO/MnO_2_ NC
Mean crystallite size (XRD), nm	37	9.84
Average particle size (TEM), nm	25–50	12–14
Surface area (BET), m^2^/g	2.246	143.65
Surface area (BJH), m^2^/g	2.246	135.73
Mean pore diameter, nm	3.85	4.42
Total pore volume, cm^3^/g	0.0095	0.194
Average pore size, nm	10.11	37.77
PZC	7.0	6.8

#### SEM and EDX

**Fig. 3:**
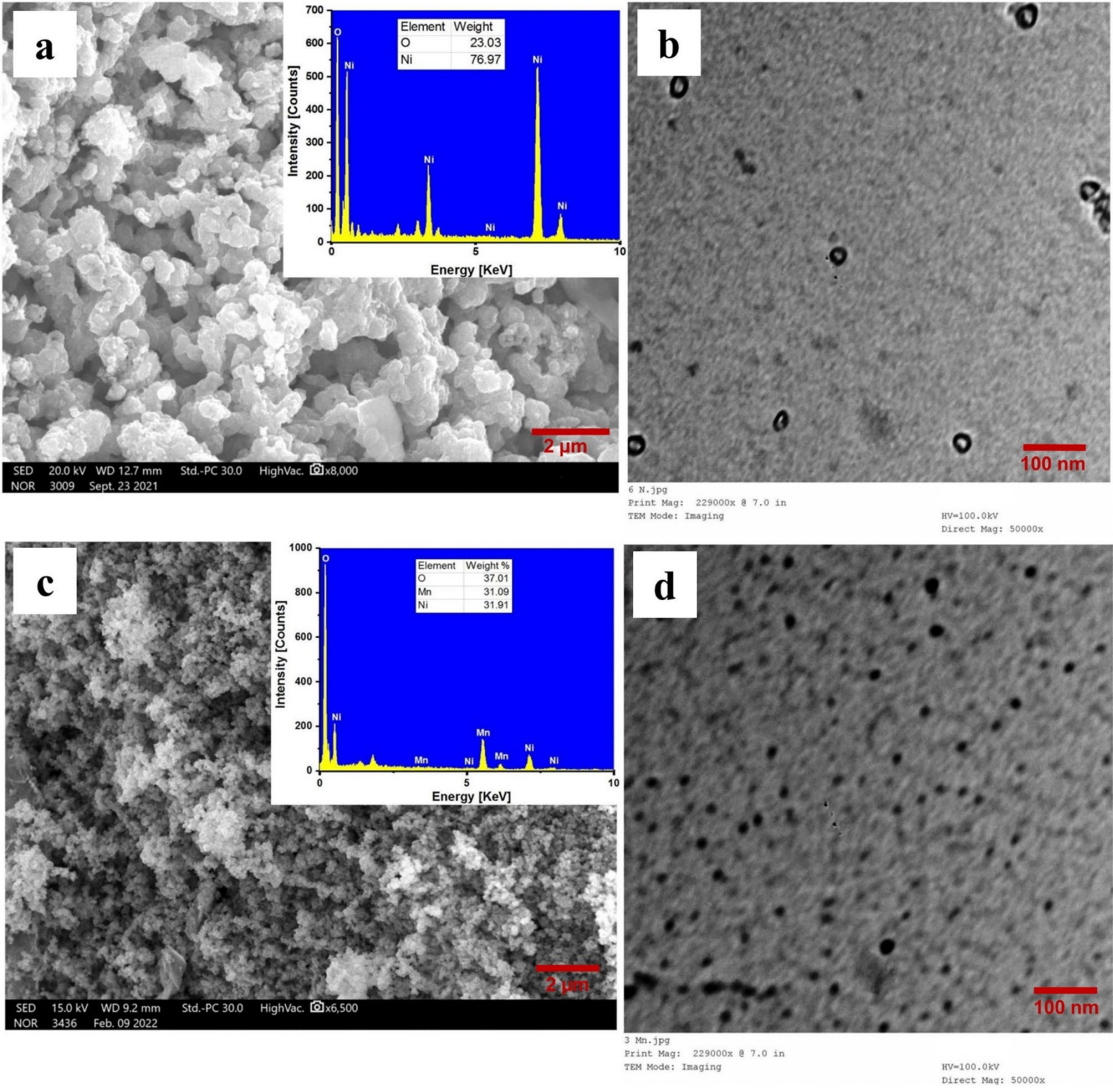
SEM (**a**), TEM (**b**) images and EDX (inset) of NiO NPs and SEM (**c**), TEM (**d**) images and EDX (inset) of NiO/MnO_2_ NC.

SEM was also utilized to identify the shape of the biosynthesized NiO NPs NiO/MnO_2_ NC. The image of NiO is displayed in Fig. 3a. This image depicts clusters of NiO NPs with closely spaced, almost spherical particles randomly orientated. The high surface energy of NiO NPs, low density, weak interparticle forces, the synthesis in an aqueous medium, and magnetic interactions between the particles are some of the possible causes of the narrow space between the particles^51,52^. The SEM image of NiO/MnO_2_ NC is shown in Fig. 3c, which exhibits identical spherical-shaped particles with uniform distribution of the nanocomposite. It can be noted that the particles of the NiO/MnO_2_ NC are much smaller than NiO NPs and much more porous, resulting in a higher surface area of the nanocomposite. The elemental composition and purity of the NiO NPs were verified by EDX analysis (inset of Fig. 3a). With a weight ratio of 76.97% Ni and 23.03% O, the EDX accurately measured the amounts of nickel and oxygen. In addition, EDX analysis clearly showed the elemental composition (O, Mn, and Ni) of the nanocomposite material with a molar ratio of 37.01% O, 31.039% Mn, and 31.91% Ni. It also showed no additional peaks resulting from impurities (inset of Fig. 3c).

#### TEM

The morphology, size, and structure of the NiO NPs were investigated using TEM studies. A TEM image of NiO NPs is presented in Fig. 3b, which verifies the production of agglomerated quasi-spherical particles with diameters ranging from 25 to 50 nm. Figure 3d displays a TEM image of the NiO/MnO_2_ NC structure. The TEM scans showed that the grains were around 12–14 nm in size and had a spherical shape (Table 1). TEM data agree with XRD and SEM data showing the superior properties of the bimetallic NiO/MnO_2_ NC over the monometallic NiO NPs, as well as the effect of *FBLE* phytochemicals in reducing metal ions to produce nanomaterials.

#### BET analysis

**Fig. 4:**
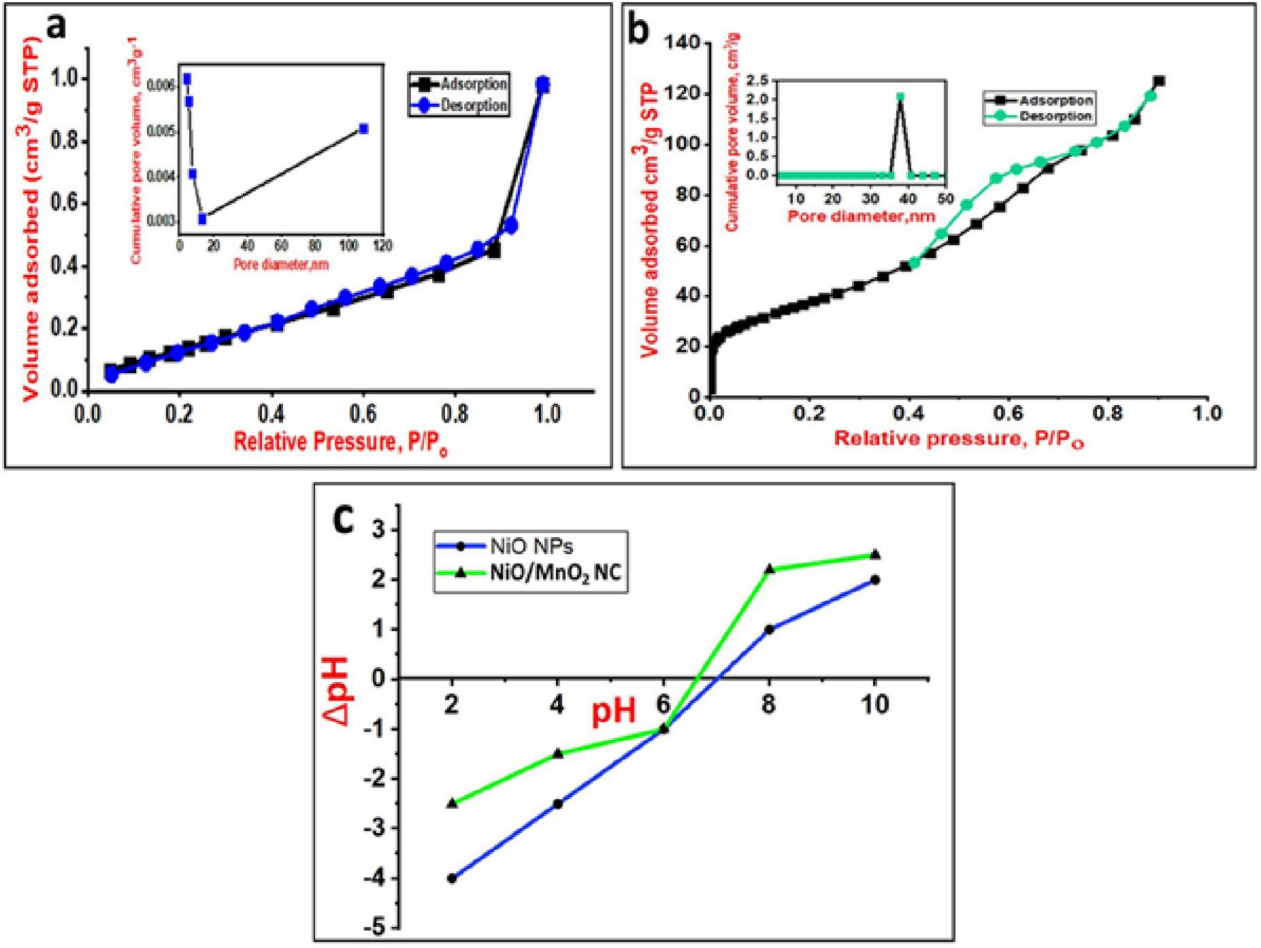
N_2_ adsorption-desorption isotherms and pore size distribution (inset) of NiO NPs (**a**) and NiO/MnO_2_ NC (**b**), and PZC of NiO NPs and NiO/MnO_2_ NC (**c**).

 Figure 4a,b display the N_2_ adsorption-desorption isotherm of the synthesized pure NiO NPs and NiO/MnO_2_ NC, respectively. The isotherm is type IV, indicating the formation of mesoporous materials. When the monolayer was completed, an indefinite multilayer was formed. Moreover, the adsorption-desorption hysteresis occurs in the p/p_0_ range of 0.78 to 0.99, which suggests materials containing mesopores^43^. These materials might be categorized as an H3-type hysteresis, and the isotherm’s loop appearance further indicates that the mesoporous NiO contains “wedge-shaped” type pores^39^. Compatibly, the sample is shown to assume surface area (S_BET_ = 3.749 m^2^/g). Additionally, the Barrett–Joyner–Halenda (BJH) (inset of Fig. 4a) theory was applied to estimate the cumulative surface area (2.25 m^2^/g), cumulative pore volume (0.0085 cm^3^/g), average pore diameter (3.85 nm), and average pore size 10.11 nm. The prepared sample for this method showed pore size larger than 2 nm. According to the BET model, the obtained NiO/MnO_2_ NC had a specific surface area of 143.65 m^2^/g. The inset of Fig. 4b shows the pore size distributions measured by the BJH model. It can be observed that the average pore size of the sample is 37.77 nm, and the mean pore dimeter is 4.42 nm. Adding a second metal in the nanocomposite can create mesopores and prevent aggregation, leading to higher surface area and mesoporosity of the NC in agreement with XRD, SEM, and TEM data^53^. In addition, the calcination step in the synthesis of NiO NPs resulted in a higher particle size and a consequent smaller surface area. This step is not required in the case of NiO/MnO_2_ NC. The high specific surface area suggested that the NiO/MnO_2_ NC may be a promising candidate for MG adsorption. Additionally, it shows that the produced particles fall within the IUPAC categorization of mesoporous solids^54^. The main analyses results are listed in Table 1.

#### PZC

The protonation-deprotonation behavior of absorbents in aqueous solutions can be characterized by the PZC^55^. The respective PZC values of NiO NPs and NiO/MnO_2_ NC were measured as 7 and 6.8, as demonstrated in Fig. 4c, and the adsorption of MG at different pH values was studied. When the the initial solution pH is lower than PZC, the surface charge of the adsorbent is positive, and vice versa. At higher pH values, the positively charged MG adsorbate and the negatively charged adsorbent surface are more attracted to each other electrostatically, leading to a higher percentage of adsorption^56^. Thus, it is expected that the cationic MG dye will favor adsorption in alkaline medium.

### Optimization of MG adsorption

#### Effect of pH

**Fig. 5:**
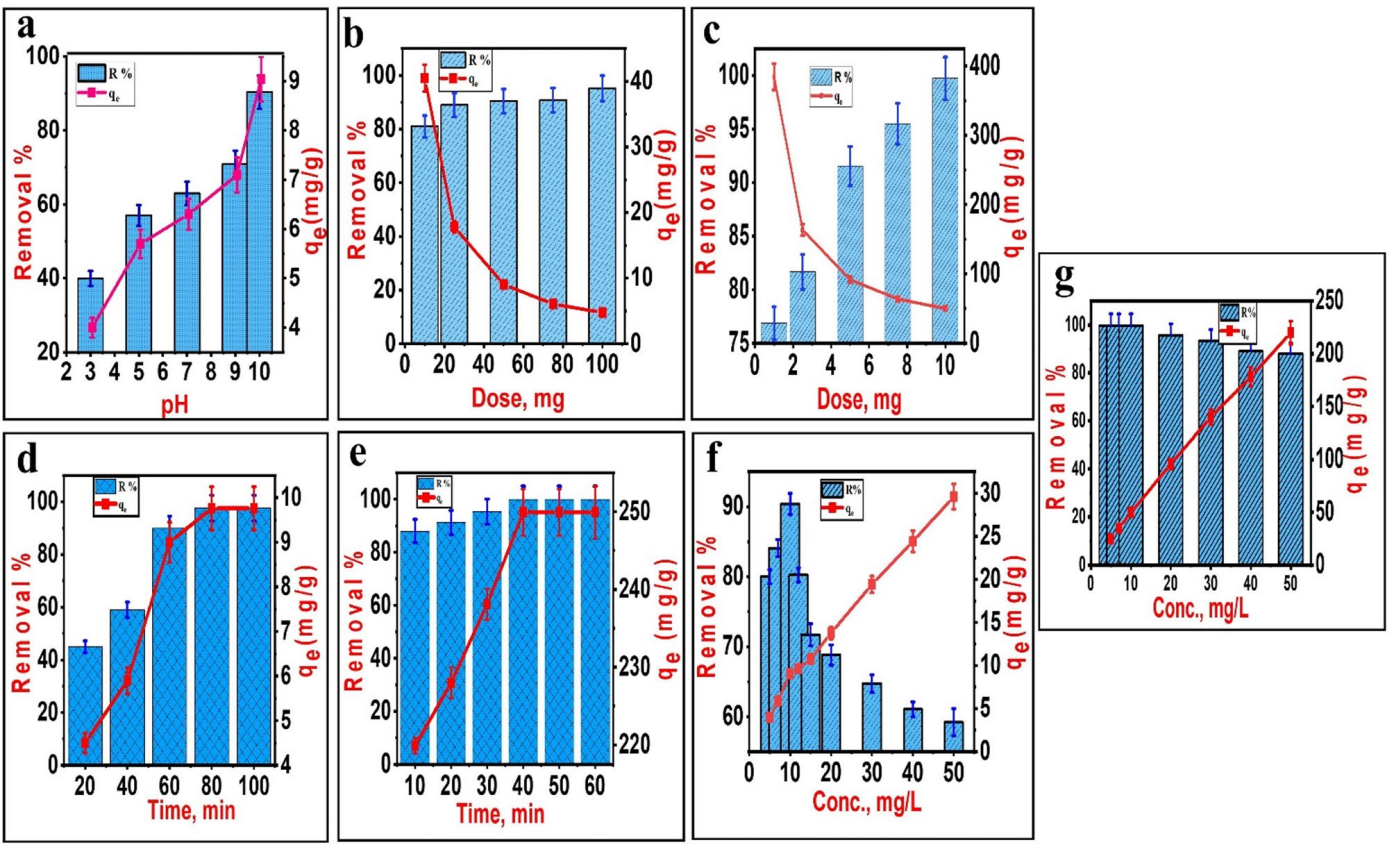
Factors affecting MG adsorption: (**a**) pH using NiO NPs adsorbent and (**b**,**c**) adsorbent dosage, (**d**,** e**) contact time, and (**f**,**g**) initial MG concentration using the respective NiO NPs and NiO/MnO_2_ NC adsorbents.

It is generally believed that the pH has a significant influence on the adsorbent’s surface charge properties and the equilibrium states of the adsorbate in the solution, both of which affect the sorption process at the solid-liquid interface^44^. As illustrated in Fig. 5a, the impact of solution pH on the NiO NPs’ capacity to remove MG dye was investigated in the pH range of 3 to 10. The findings indicated that the increase in pH from 3 to 10 led to increasing the adsorption percentage and capacity from 40 to 90.4% and from 4.0 to 9.04 mg/g, respectively. The MG removal efficiency may be discussed based on the PZC value of the NiO NPs. Decreased MG adsorption in acidic solutions when pH < PZC is caused by protonation of the functional groups on the surface of NiO NPs. This prevents positively charged MG cations from approaching the NiO NPs surface. However, at pH values greater than PZC the functional groups on NiO NPs surface are deprotonated, increasing the surface’s negative charge density and promoting MG cation binding. Due to the electrostatic attraction, the negative charge on the adsorbent surface is also helpful for adsorption, and a higher negative charge corresponds to a stronger electrostatic attraction^57^. The same discussion is applicable in case of NiO/MnO_2_ NC. As a result, a basic pH of 10 is optimal for MG adsorption as expected from PZC values and it is selected for further adsorption experiments^14^.

#### Effect of adsorbent dose

A batch adsorption system necessiates applying an optimum dosage of suitable adsorbent for maximum adsorbate removal for cost-effective application^14^. The effect of 10–100 mg doses of NiO NPs on the elimination of MG dye (10 mg/l, 50 ml) was studied at pH 10 and 25 °C. There was a noticeable increase in the percentage of MG removal by increasing NiO NPs dosage. The MG removal percentage increased sharply from 81 to 95.5% due to the higher number of available adsorption sites (Fig. 5b). However, the equilibrium adsorption capacity decreased from about 42 to 5 mg/g of NiO NPs because the increase in the adsorbent dosage for a given amount of MG resulted in the unsaturation of adsorption sites and can be further attributed to adsorbent particle aggregation^1^. Thus and from an economic point of view, a dose of 50 mg was picked for the upcoming sets. Similarly, the effect of NiO/MnO_2_ NC dosage on MG uptake was investigated from 1 to 10 mg at a fixed initial MG concentration of 10 mg/l in 50 ml solution (Fig. 5c). The percentage of MG removal was proportionally increased from 76.8 to 99.7%, and the equilibrium adsorption capacity decreased from about 100 to 86 mg/g NiO/MnO₂ NC with increasing adsorbent loading. It can be observed that NiO/MnO₂ NC yields higher equilibrium adsorption percentage and capacity values than NiO NPs. This can be attributed to the synergic effect of NiO and MnO₂ and the bimetallic nanocomposite’s extremely higher surface area than the monometallic NiO, as seen in Table 1.

#### Effect of agitation time

The duration of contact between the solid adsorbent and the adsorbate is essential for the adsorption-based treatment of water. The investigation of this characteristic can save costs and energy when it comes to industrial-scale adsorption processes^58^. Figure 5d,e present the findings of the MG adsorption rate by NiO NPs and NiO/MnO_2_ NC throughout a range of time intervals, from 20 to 100 min and from 10 to 60 min, respectively. The MG removal efficiency and adsorption capacity by NiO NPs increase from 45% to 97.6% and from 4.5 mg/g to 9.8 mg/g when the contact duration is increased from 20 to 100 min (Fig. 5d). Better results were obtained by NiO/MnO₂ NC with the increase in the MG removal efficiency from 87.95% to 99.94% and in the adsorption capacity from 219.9 to 249.9 mg/g by increasing time from 10 to only 60 min (Fig. 5e). In addition, there is no change in the removal efficiency due to reaching equilibrium after only 40 min with NiO/MnO₂ NC, but NiO needed double the time of 80 min to equilibrate. The increased MG adsorption with time was observed by other researchers^58–60^. The results further show that the uptake of MG onto the adsorbent is relatively rapid at first and then gradually slows down until reaching equilibrium. This increased initial adsorption is due to the increased number of available active surface sites on the adsorbent and the increased MG concentration gradient between the solution and the adsorbent. Laterally, the slowdown in MG adsorption was assigned to the decrease in the vacant adsorption sites and the repulsion between MG molecules in the solid and solution phases^52^. Similar trends were stated in some other publications^46–49,51^. Finally, the results show excellent adsorption performance of the prepared nanoadsorbents, especially NiO/MnO_2_ NC.

#### Effect of initial dye concentration

The effect of initial MG concentration on equilibrium adsorption was investigated at different initial concentrations and room temperature (25 °C). The results indicate that MG removal strongly depends on its initial concentration. The removal percentage of MG by NiO NPs decreased from 90.4% to 59.2%, and the capacity increased from 5.9 mg/g to 29.6 mg/g by increasing the initial concentration from 5 to 50 mg/l at constant conditions (pH = 10, time = 60 min, and dose = 50 mg), as shown in Fig. 5f. In the case of NiO/MnO_2_ NC, the adsorption percentage decreased from 99.4% to 83.2%, and the capacity increased from 24.9 mg/g to 208 mg/g with an increase in initial concentration from 5 to 50 mg/l at optimum conditions (pH = 10, time = 40 min, and dose = 10 mg) (Fig. 5g)^61^. The decrease in the removal percentages may be due to the adsorbent saturation via monolayer formation, but the increase in adsorption capacities is related to higher interaction between MG molecules and the adsorbent surface. It’s clear the better results of the nanocomposite over the single metal adsorbent.

### Adsorption isotherms

**Fig. 6:**
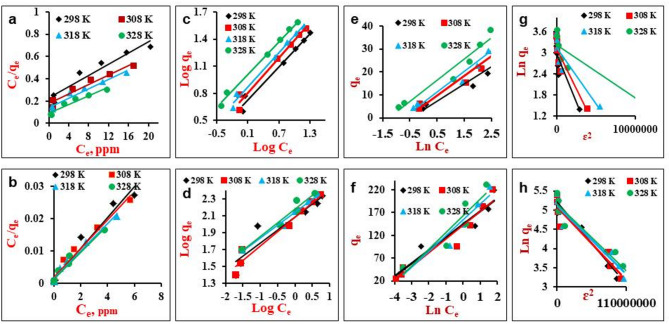
(**a**,** b**) Langmuir, (**c**,** d**) Freundlich, (**e**,** f**) Temikn, and (**g**,** h**) D-R linear plots for MG adsorption onto NiO NPs and NiO/MnO_2_ NC, respectively, at different temperatures (298, 308, 318, and 328 K).

Table 2 displays the applied model’s linear equations and their associated parameters. The linearized Langmuir, Freundlich, Temkin, and D-R plots for MG at different temperatures (298, 308, 318, and 328 K) with varying initial MG concentrations (5–50 mg/l) are presented in Fig. 6a–h and the values of the constants for each adsorption isotherm model were calculated to evaluate the surface properties and affinity limit of the adsorbent material for MG (Table 3).

**Table 2:** Linear isotherm models and their parameters used in this study.

Model	Equation	Parameters	Ref.
Langmuir	$$\begin{aligned} \:\frac{{{\mathrm{C}}_{{\mathrm{e}}} }}{{{\mathrm{q}}_{{\mathrm{e}}} }} & = \left( {\frac{1}{{{\mathrm{K}}_{{\mathrm{L}}} .{\mathrm{q}}_{{\mathrm{m}}} }}} \right) \\ & + \frac{{{\mathrm{C}}_{{\mathrm{e}}} }}{{{\mathrm{q}}_{{{\mathrm{m}}\:}} }} \\ \end{aligned}$$	q_e_ (mg/g): equilibrium adsorption capacity, q_m_ (mg/g): maximum adsorption capacity, K_L_ (l/mg): Langmuir constant; adsorption affinity, C_e_ (mg/l): equilibrium adsorbate concentration in solution	^62^
Freundlich	$$\begin{aligned} \:{\mathrm{log}}\:{\mathrm{q}}_{{\mathrm{e}}} & = \frac{1}{{\mathrm{n}}}{\mathrm{log}}\:{\mathrm{C}}_{{\mathrm{e}}} \\ & + \:{\mathrm{logK}}_{{\mathrm{F}}} \: \\ \end{aligned}$$	K_F_ (mg/g)/(mg/l)^1/n^ represents adsorption capacity, 1/n (dimensionless): Freundlich constant provides adsorption intensity	^63^
Temkin	$$\begin{gathered} q_{e} = B_{T} \ln k_{T} \hfill \\ + B_{T} \ln C_{e} \hfill \\ \end{gathered}$$	K_T_ (l/g): Temkin adsorption potential, B_T_ (J/mol): Temkin constant	^64^
D–R	$$\begin{gathered} \ln \:q_{e} \: = \:\ln \:q_{s} \hfill \\ - \:K_{{ad}} \varepsilon \:^{2} \hfill \\ \end{gathered}$$ $$\begin{gathered} \varepsilon \:\: = \:RT\ln \hfill \\ (1\: + \:1/C_{e} ) \hfill \\ \end{gathered}$$	q_s_ (mg/g): theoretical isotherm saturation capacity, K_ad_ (mol^2^/J^2^): Dubinin–Radushkevich isotherm constant, ε (J/mol): Polanyi potential, which is related to the equilibrium concentration R (8.314 J/(mol.K)): universal gas constant, T (K): temperature	^62^

The high Langmuir K_L_ values at all temperatures represent the ability of the NiO NPs and NiO/MnO_2_ NC to adsorb a significant amount of MG. Additionally, the calculation demonstrates that the maximum sorption capacities of NiO and NiO/MnO_2_ NC for MG increased with increasing temperature (Fig. 6a,b). This indicates that the adsorption is more favorable at higher temperatures. Langmuir equilibrium parameter (R_L_) is defined by Eq. (3).

3 $$\:{{R}}_{{L}}\:=\:1/\:(1\:+\:{{K}}_{{L}}{C}{o})$$

The dimensionless constant R_L_ defines the favorability of adsorption: irreversible (R_L_= 0), favorable (0 < R_L_ < 1), linear (R_L_ = 1), or unfavorable (R_L_ > 1)^57^. The R_L_ values varied from 0.01 to 0.65 for MG concentrations used in this study (5 to 50 mg/l), indicating that the adsorption of MG by NiO NPs and NiO/MnO_2_ NC was spontaneous and favorable.

Freundlich K_F_ constant refers to the adsorption intensity. The higher K_F_ value of NiO/MnO_2_ NC than NiO NPs indicates more interaction between the nanocomposite material and dye molecules^65^. As the temperature rises, n values increase as well, suggesting that adsorption is more favorable at higher temperatures (Fig. 6c,d).

The Temkin isotherm (Fig. 6e,f) considers the effect of adsorbate-adsorbent interaction on a heterogeneous surface, where the heat of adsorption decreases linearly^64^. Smaller values of the Temkin constant B_T_ for NiO NPs and NiO/MnO_2_ NC suggest that the MG adsorption on these adsorbents was favorable. The B_T_ value increased with increasing temperature, indicating endothermic adsorption as indicated in Table 3.

**Table 3:** Linear parameters of isothermal models for sorption of MG onto NiO NPs and NiO/MnO_2_ NC.

Adsorbent	T, K	Langmuir			Freundlich			Temkin		D-R			
		R^**2**^	q_**m**_, mg/g	K_**L**_, l/mg	R^**2**^	n	K_**F**_, (mg/g)/(mg/l)^1/n^	**R** ^**2**^	B_**T**_, J/mol	R^**2**^	K_**ad**_, mol^**2**^/J^**2**^	q_**s**_, mg/g	E, kJ/mol
NiO NPs	298	0.92	39.68	0.109	0.97	1.69	4.86	0.93	0.33	0.81	5*10^− 7^	18.79	1.0
	308	0.95	47.84	0.114	0.98	1.57	5.6	0.95	0.49	0.80	4*10^− 7^	20.8	1.12
	318	0.95	49.02	0.135	0.99	1.60	6.44	0.97	0.50	0.81	3*10^− 7^	21.7	1.29
	328	0.95	50.25	0.222	0.99	1.68	9.14	0.99	0.48	0.84	2*10^− 7^	24.16	1.58
NiO/MnO_**2**_ NC	298	0.97	208.3	3.70	0.89	3.22	132.9	0.95	0.086	0.95	2*10^− 8^	183.8	5.0
	308	0.97	227.3	2.30	0.96	3.00	126.9	0.94	0.084	0.91	2*10^− 8^	161.3	5.0
	318	0.98	232.56	2.87	0.98	3.28	139.5	0.92	0.077	0.92	2*10^− 8^	168.8	5.0
	328	0.97	238.1	3.23	0.96	3.10	151.0	0.88	0.074	0.92	2*10^− 8^	173.6	5.0

Compared to other models, NiO NPs results are better matched to the Freundlich isotherm (R^2^ 0.97–0.99), suggesting the formation of a multilayer adsorped on the heterogeneous surface of NiO NPs. However, the Langmuir model better fits the experimental data of MG adsorption onto NiO/MnO_2_ NC (R^2^ 0.97–0.98), referring to the adsorption of a monolayer onto the energetically homogenous surface of NiO/MnO_2_ NC. The Langmuir model has previously fitted MG adsorption by other adsorbents^66,67^.

The experimental data was also analyzed using D–R model. The β constant of the model (Fig. 6g,h) gives an idea about the mean sorption energy E. The latter provides information on the kind of adsorption technique (E < 8 kJ/mol for physical and 8 ≤ E ≤16 kJ/mol for chemisorption)^68^. It can be calculated by Eq. (4)^69^.

4 $${\text{E }} = 1/\surd 2{\mathrm{K}}_{{{\mathrm{ad}}}}$$

The magnitude of E was lower than 8 kJ/mol for NiO NPs and NiO/MnO_2_ NC at all studied temperatures (Table 3), indicating the physical nature of the adsorption process.

### Kinetic study

**Fig. 7:**
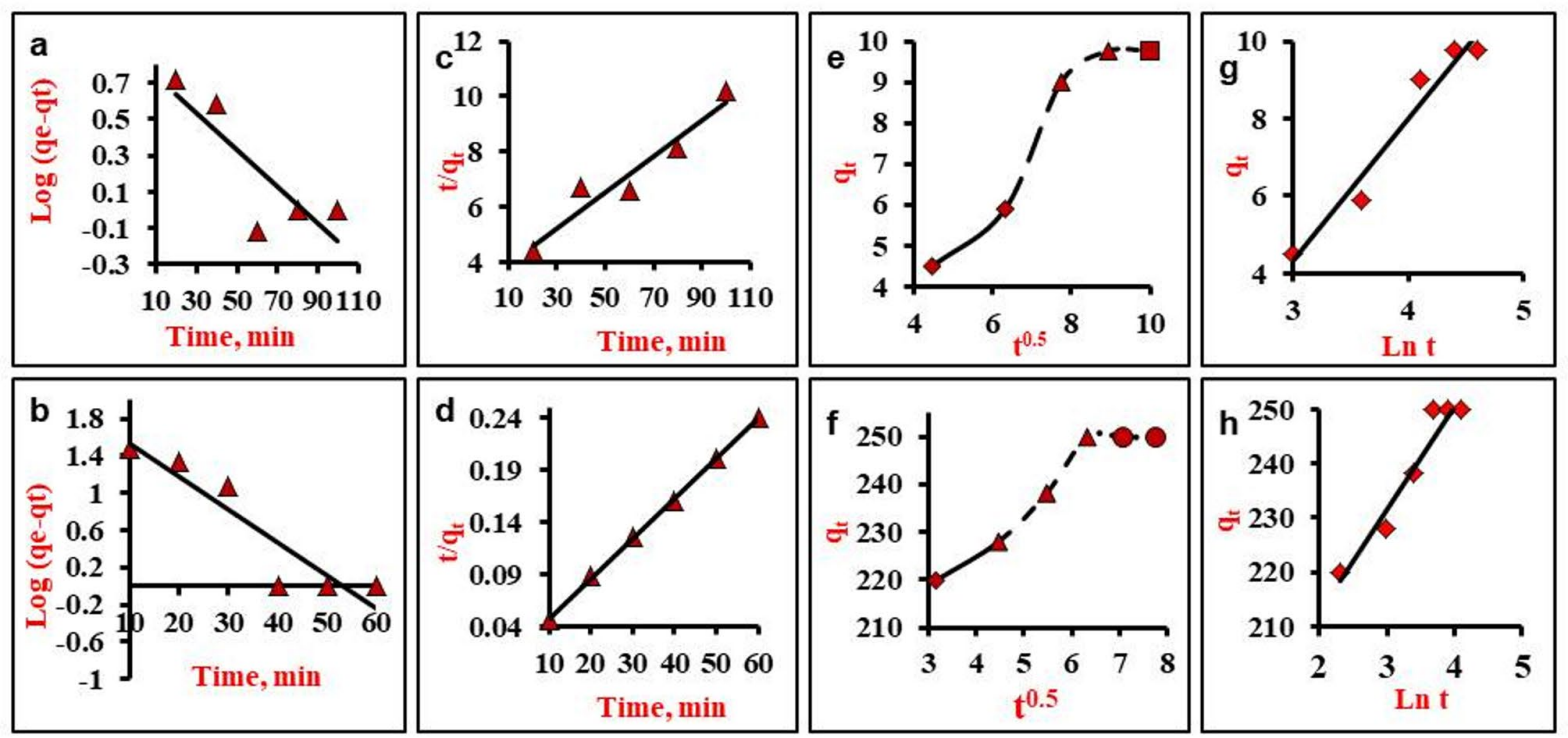
Linear plots of (**a**,** b**) PFO, (**c**,** d**) PSO, (**e**,** f**) IPD and (**g**,** h**) Elovich models for MG adsorption onto NiO NPs and NiO/MnO_2_ NC respectively at 298 K.

Four adsorption kinetic models, including PFO, PSO, IPD, and Elovich, were used to understand the primary adsorption behavior of MG (Table 4). Table 5 lists the detailed results of the four models and Fig. 7a-h exhibits the fitting curves. The PSO model (R^2^ = 0.926 and 0.99 for NiO NPs and NiO/MnO_2_ NC, respectively; Fig. 7c,d) fits the data more accurately than other models. Further, the experimental values of the equilibrium uptake q_e_ (exp.) of MG by NiO NPs and NiO/MnO_2_ NC of 15.38 mg/g and 263.15 mg/g are closer to the calculated value q_e_ (cal.) of the PSO model of 9.76 mg/g and 249.86 mg/g, respectively. Thus, the adsorption of MG on the studied nanoadsorbents is best explained by the PSO model^66,67^.These results indicate that MG uptake onto the adsorbents is probably controlled by chemical adsorption. Although kinetics are well described by PSO, isotherm models; especially the D-R energy model, indicate predominantly physical adsorption. The combined results suggest a multi-mechanistic adsorption process, with physisorption being the dominant mechanism, likely initiated by physisorption followed by chemisorption.

Notably, NiO/MnO_2_ NC has 17 times the qe value and 14 times the k_2_ value of NiO NPs, demonstrating the nanocomposite’s significantly superior adsorption efficiency. This is owing to the existence of several metal atoms, which can form unique adsorption sites with variable energies, hence improving adsorbate-adsorbent interactions^29^.

The adsorption process is further explained by IPD model as represented in Fig. 7e,f. The curves comprised three distinct sections: the diffusion of MG to the external surface of the sorbent (fluid transport), the gradual adsorption (film diffusion), and the plateau portion representing the equilibrium^70^. As can be seen, the lines do not go through the origin, suggesting some degree of boundary layer control and indicating that IPD is not the rate-controlling step in the sorption process. The results of Elovich model are shown in Fig. 7g,h; Table 5. The Elovich’s initial adsorption rate (α) is proportionated to the rate of change in chemisorption, and the desorption constant (β) is linked to the available surface sites^71^. The R^2^ values are 0.93 and 0.94 for NiO NPs and NiO/MnO_2_ NC, respectively, demonstrating the probable application of the Elovich model.

**Table 4:** Linear kinetic models applied for adsorption of MG onto NiO NPs and NiO/MnO_2_ NC.

Model	Equation	Parameters	Ref.
PFO	$$\begin{gathered} \:{\mathrm{log}}\:({\mathrm{q}}_{{\mathrm{e}}} \: - \:{\mathrm{q}}_{{\mathrm{t}}} )\: \hfill \\ = \:{\mathrm{log}}\:{\mathrm{q}}_{{\mathrm{e}}} \: - \:\left( {\frac{{{\mathrm{k}}_{1} }}{{2.303}}} \right)\:{\mathrm{t}} \hfill \\ \end{gathered}$$	q_t_ (mg/g): the amount of adsorbate adsorbed at time t, k_1_ (min^− 1^): pseudo-first-order rate constant	^72^
PSO	$\:{\mathrm{q}}_{\mathrm{t}}\:=\:\left(\frac{1}{{\mathrm{k}}_{2}{{\mathrm{q}}_{\mathrm{e}}}_{2}}\right)\:+\:\left(\frac{1}{{\mathrm{q}}_{\mathrm{e}}}\right)\:\mathrm{t}$	k_2_ [g/(mg.min)]: pseudo-second-order rate constant	
IPD	q_t_ = k_i_ t^0.5^ + C	k_i_ [mg/(g.min)^0.5^)]: intraparticle diffusion rate constant	^73^
Elovich	$q_{t} = \left(\frac{1}{\upbeta}\right)\ln(\upalpha\upbeta) + \left(\frac{1}{\upbeta}\right)\ln t$	*α* [mg/(g.min)]: the initial adsorption rate *β* (g/mg): parameter related to the extent of surface coverage and activation energy of the Elovich equation.	^57^

**Table 5:** Kinetic parameters for adsorption of MG onto NiO NPs and NiO/MnO_2_ NC.

Model	Parameters	NiO NPs	NiO/MnO_2_ NC
PFO	R^2^	0.691	0.854
	K_1_ (min^− 1^)	0.023	0.082
	q_e_ (mg/g)	7.01	151.21
PSO	R^2^	0.926	0.999
	K_2_ (g/(mg.min))	0.0197	0.273
	q_e_ (mg/g)	15.38	263.15
IPD	R^2^	0.916	0.926
	k_i_ (mg/(g.min^1/2^))	1.07	7.343
Elovich	R^2^	0.928	0.940
	α (mg/(g.min))	26.25	2*10^5^
	β (g/mg)	0.027	0.052

### Thermodynamic study

**Fig. 8:**
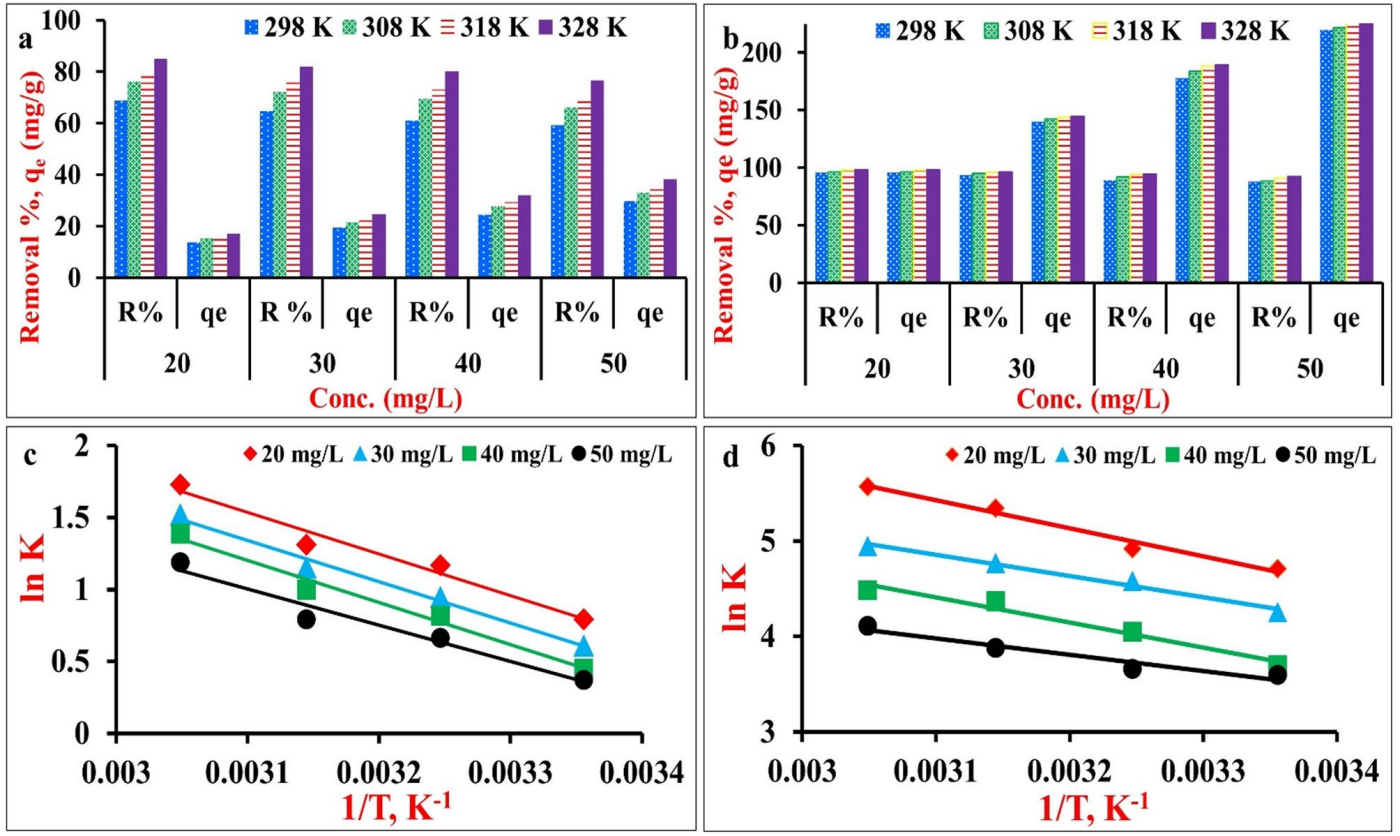
(**a**,** b**) Effect of temperature and adsorption capacity, and (**c**,** d**) Van’t Hoff plot of MG adsorption onto NiO NPs and NiO/MnO_2_ NC, respectively, with different concentrations.

The effect of temperature on the adsorption percent and capacity of MG dye onto NiO NPs and NiO/MnO_2_ NC is investigated in the range 298–328 K and the results are drawn in Fig. 8a,b. All the values of removal perecent R% and adsorption capacity qe increased with the increase in temperature from 298 K to 328 K at all studied concentrations, indicating that the process is endothermic^74^. This may be attributed to the increase in the diffusion rate of MG dye molecules across the the external boundary layers to the internal pores of the adsorbent due to the decrease in the solution viscosity at high temperatures^75^. Also, more dye molecules may have sufficient energy to interact with active sites on the adsorbent surface.

To assess the thermodynamic feasibility and spontaneity of the MG adsorption, the standard adsorption thermodynamic parameters: standard enthalpy (ΔH^o^), standard entropy (ΔS^o^), and standard free energy (ΔG^o^) were determined (Table 6). The values of ΔH^o^ and ΔS^o^ were calculated from the slope and the intercept of the Van’t Hoff linear Eq. (5) as represented in Fig. 8c,d, and the distribution coefficient K_C_ was determined using Eq. (6).

5 $$\:{l}{n}\:{{K}}_{{C}}\:=\:\frac{{\Delta\:}{{S}}^{{o}}}{{R}}-\:\frac{{\Delta\:}{{H}}^{{o}}}{{R}{T}}$$

6 $$\:{{K}}_{{C}}\:=\frac{{{q}}_{{e}}}{{{C}}_{{e}}}$$

where q_e_ is the equilibrium uptake of MG by NiO NPs or NiO/MnO_2_ NC (mg/g), and C_e_ is the equilibrium concentration in solution (mg/l).

Figure 8a,b show that the adsorption process is temperature sensitive for both adsorbents across the measured range of MG concentrations, as R% and q_e_ values rise with increasing temperature. The ranges of R^2^ values of Van’t Hoff plots were 0.96–0.99 for NiO NPs and 0.94–0.98 for NiO/MnO_2_ NC. The ΔH^o^ has positive values (from 14.19 to 24.35 kJ/mol), less than 80 kJ/mol over the studied concentration range, confirming the endothermic nature and the physical adsorption of MG onto NiO NPs and NiO/MnO_2_ NC, in agreement with the favorable adsorption at higher temperatures. The ΔS^o^ values were also positive, demonstrating the increment in the degrees of freedom during the MG sorption process^69^. Further, the ΔG^o^ values were calculated using Eqs. (7) and (8) (R is the universal constant 8.314 J/mol/K and T is the temperature in K) and the results are listed in Table 6.

7 $$\:{\Delta\:}{G}^\circ\:\:=\:{\Delta\:}{{H}}^{{o}}\:-\:{T}{\Delta\:}{{S}}^{{o}}$$

8 $$\:{\Delta\:}{G}^\circ\:\:=\:-\:{R}{T}\:{l}{n}\:{{K}}_{{C}}$$

The values of ΔG^o^ were negative at all temperatures and concentrations, confirming the feasibility and the spontaneous nature of MG adsorption onto NiO NPs and NiO/MnO_2_ NC adsorbents. The increase in the absolute ΔG^o^ values with the temperature rise at all measured MG concentrations indicates the higher affinity of the two adsorbents for MG at higher temperature^74^. NiO/MnO_2_ NC has much larger ΔG^o^ values than NiO NPs, indicating superior adsorption efficiency in agreement with isotherm and kinetic results, as previously discussed. Finally, the adsorption behavior of the green-synthesized nanoadsorbents indicates a multi-mechanistic adsorption route with physisorption as the dominating step, followed by partial chemisorption at certain active sites^76,77^.

**Table 6:** Thermodynamic parameters for MG adsorption onto NiO NPs and NiO/MnO_2_ NC.

C_ο_, mg/l	Temp., K	NiO NPs			NiO/MnO_2_ NC		
		ΔG^o^, kJ/mol	ΔH^o^, kJ/mol	ΔS^o^, kJ/(mol.K)	ΔG^o^, kJ/mol	ΔH^o^, kJ/mol	ΔS^o^, kJ/(mol.K)
20	298	− 1.963	24.002	0.087	− 11.673	24.389	0.121
	308	− 2.990			− 12.595		
	318	− 3.467			− 14.135		
	328	− 4.718			− 15.191		
30	298	− 1.505	23.844	0.085	− 10.541	18.502	0.098
	308	− 2.425			− 11.721		
	318	− 3.041			− 12.612		
	328	− 4.143			− 13.589		
40	298	− 1.114	24.337	0.085	− 9.163	21.872	0.104
	308	− 2.092			− 10.374		
	318	− 2.635			− 11.557		
	328	− 3.788			− 12.228		
50	298	− 0.925	20.910	0.073	− 8.913	14.190	0.077
	308	− 1.698			− 9.377		
	318	− 2.094			− 10.256		
	328	− 3.247			− 11.211		

### Influence of interfering ions

**Fig. 9:**
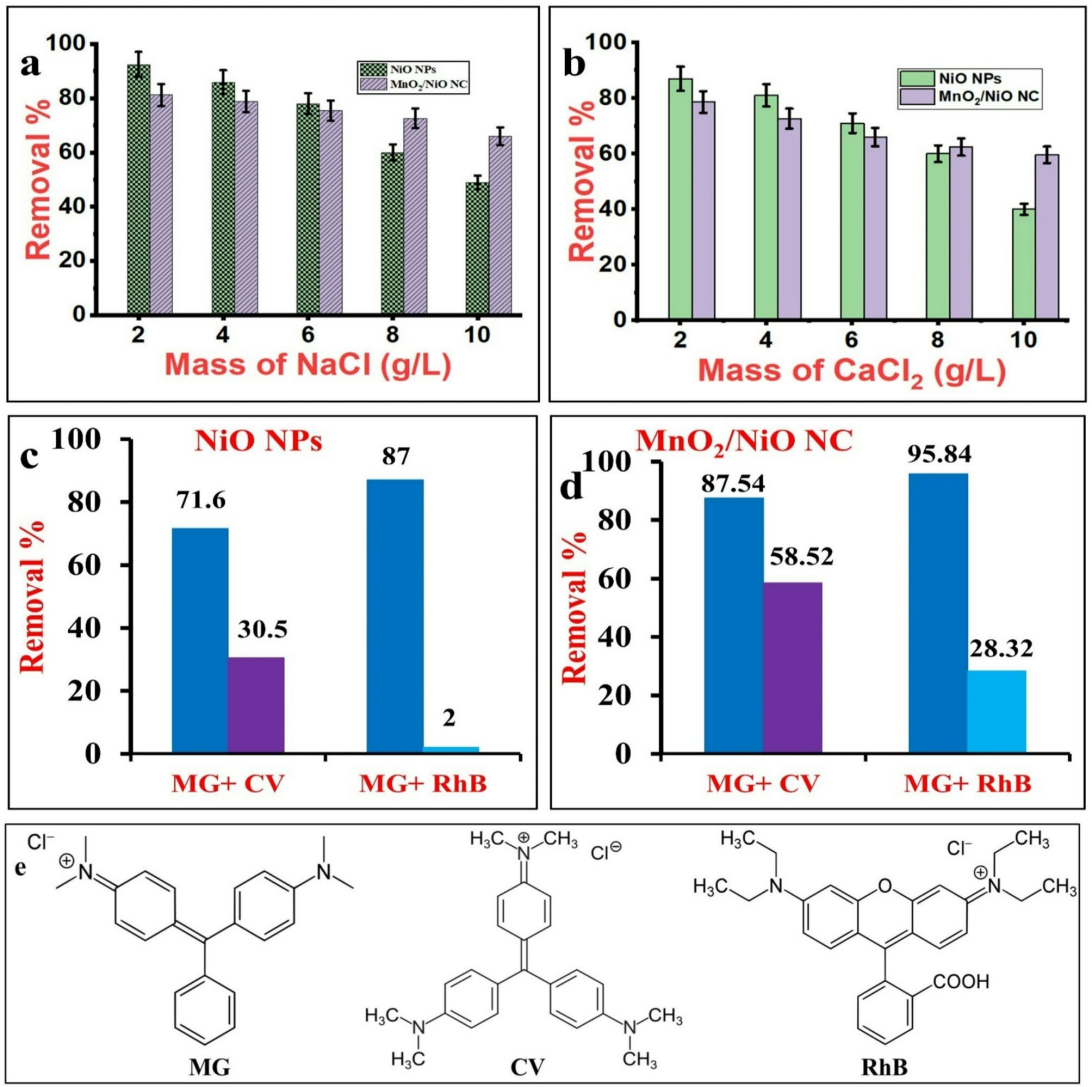
Effect of different concentrations of (**a**) NaCl and (**b**) CaCl_2_ on the adsorptive performance of NiO NPs and NiO/MnO_2_ NC, adsorption efficiency of MG from binary mixtures with CV or RhB by (**c**) NiO NPs and (**d**) NiO/MnO_2_ NC, and (**e**) structures of the three dyes.

The dye industry wastewater contains a lot of inorganic cations that create colloidal instability by reducing the interaction between the target dye molecules and the adsorbent surface^75^. Thus, the impact of dissolved Na^+^ and Ca^2+^ cations on MG adsorption by NiO NPs and NiO/MnO_2_ NC has been examined, and the results are shown in Fig. 9a,b, respectively. The MG removal efficiency on NiO NPs decreased by 47% (from 92.5 to 49%) and on NiO/MnO_2_ NC by 19% (from 81.2 to 66%) when the Na^+^ concentration was increased from 2 to 10 g/l (Fig. 9a). A higher decrease occurred in MG removal of 54% on NiO NPs (from 87 to 40%) and 24% on NiO/MnO_2_ NC (from 78.6 to 59.6%) by increasing Ca^2+^ concentration from 2 to 10 g/l (Fig. 9b). This decrease in MG uptake is attributed to the competitive adsorption between the cationic MG molecules and the cations present in the solution for the negatively charged binding sites on the surface of the adsorbent. Additionally, the lower adsorption efficiency in the presence of the divalent Ca^2+^ than the monovalent Na^+^ at the same concentration since the former blocking more binding sites than the latter^78^. Further, the NiO/MnO_2_ NC is more stable than NiO NPs, as evidenced by a smaller drop in MG removal by the former.

### Effect of competing dyes on adsorption

The results of binary dyes adsorption are shown in Fig. 9c,d. The MG percent removal was 71.65% and 87.4% by NiO NPs, and 87.54% and 95.89% by NiO/MnO_2_ NC in the presence of CV and RhB, respectively. This indicates the higher adsorption capacity of NiO/MnO₂ NC over NiO NPs in binary mixtures. On the other hand, the percent removal of CV and RhB was 28.86% and 1.7% by NiO NPs and 58.52% and 26.81% by NiO/MnO₂ NC, demonstrating the superior adsorption of MG by the two adsorbents. This selectivity is less pronounced by NiO/MnO₂ NC, probably due to the diverse surface sites resulting from the presence of two different metals that create varying energy adsorption sites. However, this property is important to simultaneously remove different pollutants with diverse structures.

The selective adsorption of the triphenyl methane MG dye in the presence of competing triphenyl methane CV dye and xanthene RhB dye was also investigated by calculating the selectivity coefficient (k_S_) by Eq. (9).

9 $$\:{{k}}_{{S}}\:=\:{{K}}_{{d}}\left({M}{G}\right)/{{K}}_{{d}}\left({d}{y}{e}\right)$$

where K_d_ is the distribution coefficient, which refers to the binding affinity of MG to NiO NPs or NiO/MnO_2_ NC and can be calculated from Eq. (10):

10 $$\:{{K}}_{{d}}=\:\left(\frac{{V}}{{W}}\right).\left(\frac{{{C}}_{0}\:-\:{{C}}_{{e}}}{{{C}}_{{e}}}\right)$$

The calculated selectivity coefficients of MG in the presence of CV and RhB are listed in Table 7. The high k_S_ values suggest a higher selectivity of MG adsorption by NiO NPs and NiO/MnO_2_ NC. Furthermore, the lower adsorption efficiency for RhB can be attributed to its different chemical structure (Fig. 9e) and the more significant adsorption steric hindrance during the transfer of dye molecules to the pores of the adsorbent^79^.

**Table 7:** Parameters of adsorption selectivity in binary mixtures.

Binary mixture	Dye	NiO NPs		NiO/MnO_2_ NC	
		K_d_	k_s_	K_d_	k_s_
MG/CV	MG	25.27	6.23	70.25	4.98
	CV	4.06		14.11	
MG/RhB	MG	69.37	399.94	233.23	63.68
	RhB	0.17		3.66	

### Reusability

**Fig. 10:**
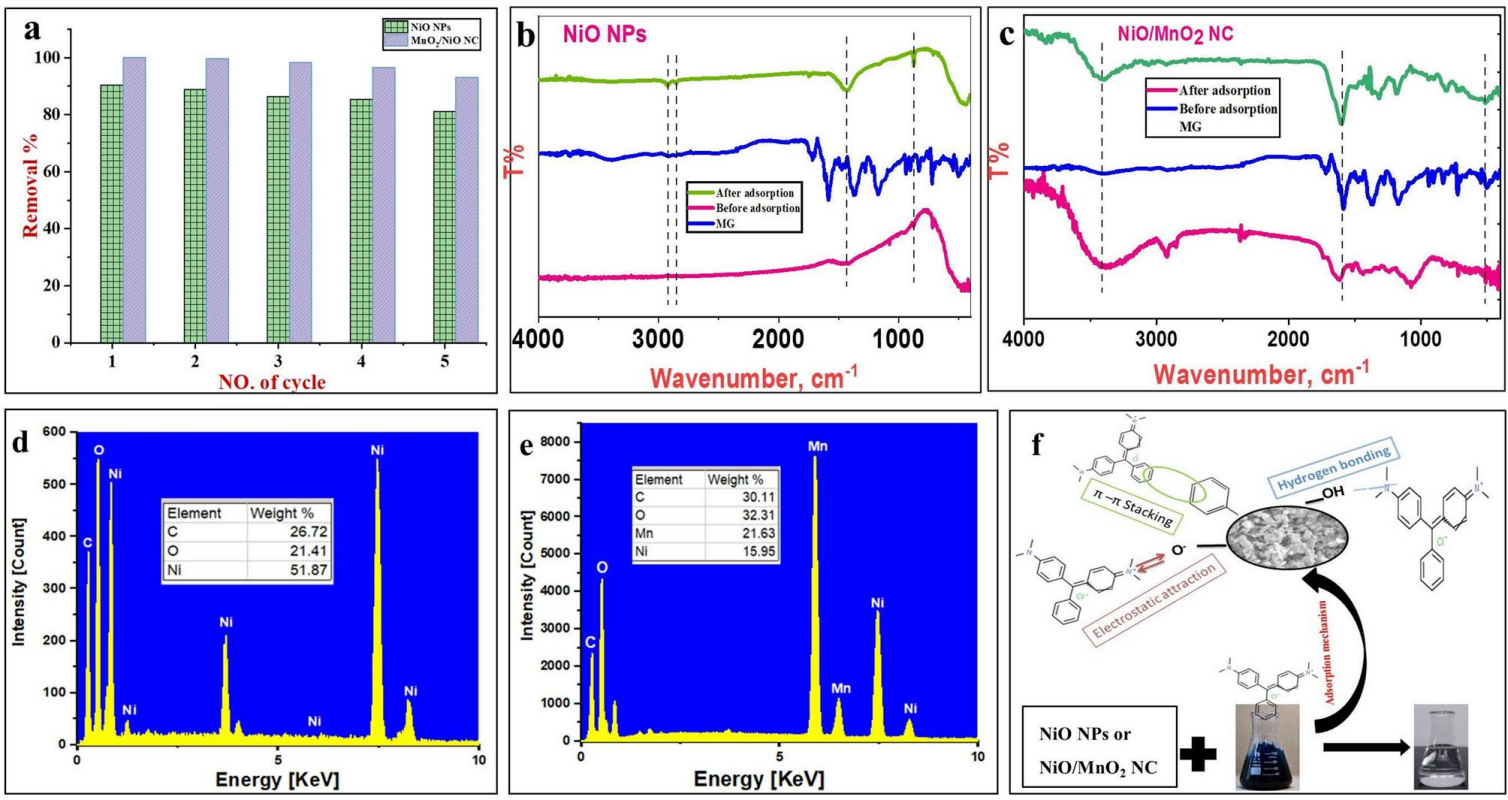
(**a**) Removal efficiency of MG onto NiO NPs and NiO/MnO_2_ NC after five cycles, FTIR spectra of (**b**) NiO NPs and (**c**) NiO/MnO_2_ NC before and after MG adsorption, EDX profiles of NiO NPs (**d**) and NiO/MnO_2_ NC (**e**) after MG adsorption, and (**f**) MG adsorption mechanism.

Adsorbent regeneration is essential for cost-effective and practical green applications. Thus, the stability and reusability of the adsorbents towards MG adsorption were evaluated at optimum conditions, as presented in Fig. 10a. After the fifth run, the results clarified that the MG removal efficiency was slightly decreased by 10.4% (from 90.4% to 81.0%) onto NiO NPs and by 6.9% (from 99.94% to 93.07%) onto NiO/MnO_2_ NC, showing the good stability of the prepared adsorbents. Additionally, the stability and reusability of the bimetallic adsorbent outperform the monometallic one, probably due to the lower aggregation in the presence of two different metal atoms. It should be noted that the regeneration using ethanol and bidistilled water may contribute to secondary pollution by releasing traces of the adsorbed dye into the aqueous phase, despite the high reusability of NiO and NiO/MnO_2_ nanocomposites for MG dye adsorption. Several environmentally friendly tactics should be considered to mitigate this risk, including recycling the washings to recover MG dye, utilizing closed-loop regeneration systems, and employing mild photocatalytic or thermal regeneration procedures that promote dye degradation rather than release. Furthermore, during regeneration, the use of advanced oxidation processes may efficiently break down any remaining organic contaminants. To guarantee the sustainability and safety of the regeneration process for the environment, future studies should concentrate on empirically testing these strategies.

### Adsorption mechanism: systematic data and discussion

FTIR and EDX analyses were performed to elucidate the removal mechanism. FTIR spectra of adsorbents before and after the MG adsorption are compared (Fig. 10b,c). For NiO NPs, new peaks have appeared after MG adsorption (Fig. 10b). These include characteristic peaks at 2926 and 2848 cm^− 1^ that are assigned to C–H (aromatic) stretching vibrations and at 870 cm^− 1^ due to the aromatic C=C binding of MG^80^. Additionally, the C–H bending peak intensity at 1452 cm^− 1^ increases after adsorption due to MG adsorption. Similarly, some of the characteristic peaks of MG are found in the MG-loaded NiO/MnO_2_ NC, which are absent in the pure NiO/MnO_2_ NC, confirming the existence of MG. Furthermore, the 3360 cm^− 1^ and 552 cm^− 1^ peaks have noticeable shifts to 3440 and 570, respectively. In addition, the FTIR spectrum shows the absorption band at 1600 cm^− 1^, which is attributed to a C=C bond (Fig. 10c).

The adsorbent’s EDX profiles before and after MG adsorption are compared to further examine the removal mechanism. The profiles of adsorbents show the carbon signal on the surface of the adsorbent after adsorption, confirming the successful loading of MG (Fig. 10d,e). The NiO/MnO_2_ NC’s large surface area and abundant surface hydroxyl groups support an initial quick physisorption stage followed by a delayed chemisorption phase occurs when MG functional moieties interact with metal oxide sites (Ni^2+^/Mn^4+^) through surface complexation or coordination^76,77^. Upon these results and isotherm and kinetic analyses, MG molecules can be adsorbed onto the adsorbents via different interactions, such as electrostatic interactions between the deprotonated hydroxyl groups on the adsorbent surface (when pH > PZC) and the positively charged MG cations, H-bonding between the hydrogen atoms of hydroxyl groups of the adsorbents and electron-rich atoms in MG, and π-π interaction between aromatic groups in adsorbent surfaces and MG molecules, as shown in Fig. 10f.

### Comparison with other adsorbents

The optimum conditions and the adsorption capacity of MG by some previously reported adsorbents are summarized in Table 8. The biosynthesized NiO NPs and NiO/MnO_2_ NC showed higher adsorption capacity than most reported adsorbents in the literature. Our adsorbents were mainly synthesized using low-cost and ecofriendly methods utilizing FB leaf extract. In addition, the synthesized adsorbents have superior properties such as small particle size, high surface area, richness in surface acidic oxygen groups, and mesoporosity with large pore volume, providing more available active sites and improved interaction probabilities for adsorption. Moreover, dye molecules might easily penetrate and lodge onto the channel textures. Another advantage of these adsorbents is their MG selective adsorption. Thus, our adsorbents are promising for the selective adsorption of MG dye from various aqueous media.

**Table 8:** MG adsorption conditions and capacities using some adsorbents.

Adsorbent	pH	MG conc., mg/l	Equilibrium time, min	Dose, g	Adsorption capacity, mg/g	Adsorption isotherm	Temp., ◦C	Ref.
MnO_2_ NPs	10	10	60	0.01	188.8	Langmuir	25	^14^
ZIF-8@Fe/Ni	4.5	50	30	0.5	4.93	Freundlich	25	^70^
Calcium silicate nanopowders	6.5	10	60	0.5	19.12	Freundlich	30	^80^
CoO NPs	7	40	120	0.12	238.10	Langmuir	–	^81^
Fe-doped Al_2_O_3_	10	100	60	0.5	52.63	Freundlich	25	^82^
^a^GO@ZnO-NiFe_2_O_4_-α-Al_2_O_3_ nanocomposites	7	500	120	–	607	Langmuir	25	^83^
^b^ZnO-NRs-AC	–	10	3	0.035	59.17	Langmuir	–	^84^
Chitosan–zinc oxide composite	8	2.3	180	0.6	11	Langmuir	25	^85^
ZIF-8@Fe/Ni nanocomposite	4.5	50	120	0.5	151.520	Freundlich	45	^86^
^c^Fe_3_O_4_/MWCNT	6	–	80	0.01	55.25	Langmuir	25	^87^
Mn_3_O_4_ NPs	5	–	20	0.15	122	Langmuir	–	^88^
NiO NPs	10	10	60	0.05	39.68	Freundlich	25	Present work
NiO/MnO_2_ NC	10	50	60	0.01	208.3	Langmuir	25	

^a^Nickel ferrite, zinc oxide, and alpha alumina nanoparticles onto graphene oxide.

^b^ZnO nanorod-loaded activated carbon.

^c^Magnetite decorated multi-walled carbon nanotubes.

## Conclusions

In this work, NiO NPs and NiO/MnO_2_ NC were successfully synthesized using natural compounds extracted from Ficus benjamina leaves for the removal of MG dye from water. UV-Vis, TAG, FT-IR, XRD, SEM, EDX, and N_2_ adsorption-desorption isotherm measurements were used to thoroughly characterize the green adsorbents that were produced. MG was most effectively adsorbed at a pH of 10, a 60-min contact period, and a 10 mg adsorbent dosage in the case of NiO/MnO_2_ NC and 50 mg in the case of NiO NPs. Moreover, in the case of NiO NPs, the MG adsorption isotherm was more closely related to the Freundlich model, while in the case of NiO/MnO_2_ NC, it was closer to the Langmuir.The maximum adsorption capacities of MG by NiO NPs and NiO/MnO_2_ NC were calculated as 39.68 mg/g and 208.30 mg/g, respectively. In addition, thermodynamic studies showed positive values of ΔS^o^, suggesting an increase in randomness at the solution/solid interface, and positive values of ΔH^o^, suggesting an endothermic adsorption process. Further, the negative values of ΔG^o^ confirmed that the adsorption process is spontaneous. Although PSO accurately describes kinetics, isotherm models, particularly the D-R energy model, show that physical adsorption is the primary mechanism. The combined data point to a multi-mechanistic adsorption process, with physisorption being the primary mechanism. Combined results indicate that the adsorption mechanism is based on physical and chemical interactions between the functional groups of the adsorbents and the MG molecules, such as electrostatic interaction, hydrogen bonding, and π-π interaction. All adsorbents could be regenerated five times with a slight decrease in removal efficiency. The synergistic effect of the bimetallic nanocomposite results in its better removal efficiency and stability than the monometallic nanoparticles. Finally, developed adsorbents are cheap and effective for removing cationic dyes from wastewater.

## Data Availability

The datasets generated and analyzed during the current study are available from the corresponding author upon reasonable request.

## Code availability

No custom codes were used in this study.
